# Characterization of the Transient Deficiency of PKC Isozyme Levels in Immature Cord Blood T Cells and Its Connection to Anti-Allergic Cytokine Profiles of the Matured Cells

**DOI:** 10.3390/ijms222312650

**Published:** 2021-11-23

**Authors:** Khalida Perveen, Alex Quach, Michael J. Stark, Susan L. Prescott, Simon C. Barry, Charles S. Hii, Antonio Ferrante

**Affiliations:** 1 Department of Immunopathology, SA Pathology at the Women’s and Children’s Hospital, North Adelaide, SA 5006, Australia; khalida.perveen@adelaide.edu.au (K.P.); Alexander.Quach@sa.gov.au (A.Q.); charles.hii@adelaide.edu.au (C.S.H.); 2 Adelaide School of Medicine and the Robinson Research Institute, University of Adelaide, Adelaide, SA 5005, Australia; Michael.stark@adelaide.edu.au (M.J.S.); simon.barry@adelaide.edu.au (S.C.B.); 3 Department of Neonatal Medicine, Women’s and Children’s Hospital, North Adelaide, SA 5006, Australia; 4 School of Paediatrics and Child Health, University of Western Australia, 35 Stirling Highway, Perth, WA 6009, Australia; Susan.Prescott@telethonkids.org.au; 5 The ORIGINS Project, Telethon Kids Institute and Perth Children’s Hospital, 15 Hospital Avenue, Nedlands, WA 6009, Australia; 6 School of Biological Sciences, University of Adelaide, Adelaide, SA 5005, Australia

**Keywords:** neonate, cord blood T cells, CD4^+^ and CD8^+^ T cells, T cell maturation, Th1 and Th2/9 subsets, PKC isozymes, PKCζ, cytokines, allergy

## Abstract

Cord blood T cells (CBTC) from a proportion of newborns express low/deficient levels of some protein kinase C (PKC) isozymes, with low levels of PKCζ correlating with increased risk of developing allergy and associated decrease in interferon-gamma (IFN-γ) producing T cells. Interestingly, these lower levels of PKCζ were increased/normalized by supplementing women during pregnancy with n-3 polyunsaturated fatty acids. However, at present, we have little understanding of the transient nature of the deficiency in the neonate and how PKCζ relates to other PKC isozymes and whether their levels influence maturation into IFN-γ producing T cells. There is also no information on PKCζ isozyme levels in the T cell subpopulations, CD4^+^ and CD8^+^ cells. These issues were addressed in the present study using a classical culture model of neonatal T cell maturation, initiated with phytohaemagglutinin (PHA) and recombinant human interleukin-2 (rhIL-2). Of the isozymes evaluated, PKCζ, β2, δ, μ, ε, θ and λ/ι were low in CBTCs. The PKC isozyme deficiencies were also found in the CD4^+^ and CD8^+^ T cell subset levels of the PKC isozymes correlated between the two subpopulations. Examination of changes in the PKC isozymes in these deficient cells following addition of maturation signals showed a significant increase in expression within the first few hours for PKCζ, β2 and μ, and 1–2 days for PKCδ, ε, θ and λ/ι. Only CBTC PKCζ isozyme levels correlated with cytokine production, with a positive correlation with IFN-γ, interleukin (IL)-2 and tumour necrosis factor-alpha (TNF), and a negative association with IL-9 and IL-10. The findings reinforce the specificity in using CBTC PKCζ levels as a biomarker for risk of allergy development and identify a period in which this can be potentially ‘corrected’ after birth.

## 1. Introduction

Protein kinase C (PKC) is composed of three subfamilies of protein kinases, namely, the classical (α, β1, β2, γ), novel (δ, μ, ε, θ, η) and atypical (ζ, ι/λ) PKC isozymes. The classical isozymes require calcium, diacylglycerol and phospholipids for activation whereas the novel isozymes require only diaylglycerol and phospholipids. Unlike the classical and novel isozymes, the atypical PKC family members, lacking functional C2 and C1 domains, require only phospholipids for activation [1,2]. Amongst the atypical PKC isozymes, PKC ι/λ is generally more abundantly expressed and with a wider tissue distribution than PKCζ [3]. However, brain, lung and testes have a higher content of PKCζ than PKCλ/ι. It has been demonstrated that the expression of the atypical PKC isozymes is dysregulated in cancer. Thus, in most cancers, except in melanoma, PKCλ/ι expression is upregulated. Similarly, PKCζ expression is also upregulated in many cancers but downregulated in cancer of the brain, lung and testes, the tissues in which its expression is higher than that of PKCλ/ι [3].

The atypical PKC isozymes have been reported to be involved in regulating cell polarity, growth of cancer cells, asymmetric cell division and cell-fate determination [3]. PKCζ has been reported to promote cell survival via the phosphorylation of Ser311 of RelA, a key member of the NF-κB transcription factor [4]. Our studies in cord blood T cells (CBTCs) also support a prosurvival role of PKCζ as knockdown of the isozyme decreases cell survival [5]. In B cells, PKCζ signalling is important for B cell activation and survival [6]. In CD8^+^ T cells, atypical PKC isozymes have been reported to regulate asymmetric division in fate specification that leads to the development of effector and memory cells. Deletion of either PKCζ or PKCι/λ, skews development towards the effector lineage with a resultant deficit of memory cells [7]. Interestingly in mice, PKCζ has been reported to promote the differentiation of murine T cells along the T helper 2 (Th2) lineage as loss of the isozyme results in a severe loss of activation of transcription factors such as GATA3, Stat6 and NFATc1 that are essential for Th2 polarisation [6]. The role of PKCζ in Th cell differentiation is clear different between mice and human.

The perinatal period is characterised by an adaptive Th2 cytokine bias, reflecting the immunomodulatory milieu of pregnancy. Thereafter, normal immune maturation depends on effective switching to more mature and regulated Th1 responses. Failure to do so, with the continual propensity for a Th2 functional phenotype, is associated with predisposition for allergic responses. The molecular basis for this continued Th2 propensity, while of fundamental importance, remains ill-defined. Our work has demonstrated that CBTC from babies whose mothers have a family history of allergy express low PKCζ [8]. There was a correlation between these levels in immature CBTC and the development of allergic sensitization at the age of 1–2.5 years. It was also evident that low levels of PKCζ in CBTCs correlated with the development and maturation of cells with a propensity to display Th2 cytokine bias [9]. We have also shown that knocking down PKCζ in CBTC was associated with their development towards a Th2 bias [5]. Together, the data suggest that the PKCζ level in immature T cells at birth may be a key determinant in allergy development later in life, either as a consequence of modern environmental changes (declining microbial diversity, pro-inflammatory dietary patterns and environmental toxins) or by creating greater vulnerability to their effects.

We have previously demonstrated that supplementation of women during pregnancy with n-3 polyunsaturated fatty acids or fish oil leads to an increase in the levels of PKCζ expression in CBTCs expressing low levels of this isozyme. Since these PKC isozyme levels increase/normalise during maturation [10,11,12] it is important to characterise the period of low expression since this seems to be a determinant for development towards an allergy cytokine pattern [5,8,10]. In this concept, it is proposed that the levels of PKCζ prior to T cells being engaged by the maturation agents could be critical to their development to cells with a propensity to produce anti-allergy promoting cytokines. Consequently, it is important to identify if a ‘time window’ of opportunity exists for potential environmental/epigenetic intervention to modify the pre-maturation signalling of the T cells.

The deficiency in PKC isozymes was also reported for splenic T cells from 1-day old mouse pups which normalized by 28 days [11]. However, at present, we know very little about the kinetics of PKCζ increases during the neonatal period. Identification of this “early critical window” of development may assist in the development of strategies to alter the risk of subsequent sensitization and disease, such as through nutritional supplementation [13] and other environmental strategies. Furthermore, we know even less about the state of other PKC isozymes in relation to such T cell development and kinetics of changes during neonatal T cell maturation. In addition, previous work did not examine the PKC isozyme levels in the CD4^+^ and CD8^+^ T cell subpopulations. Therefore, the aim of the current study was to examine CD4 and CD8 CBTC PKCζ levels and whether other PKC isozyme levels influence maturation towards interferon-gamma (IFN-γ) producing T cells. Furthermore, the transient state of the PKC isozyme deficiency will be defined, following maturation signal activation.

## 2. Results

### 2.1. PKC Isozyme Expression in CB CD3^+^ T Cells

Experiments were conducted with whole blood assays using anti-CD3 monoclonal antibody to gate on CD3^+^ T cells and anti-PKC isozyme specific monoclonal antibodies to detect PKC isozymes by intracellular staining and flow cytometry analysis [12]. To ensure consistency between different runs a standard cryopreserved PBMC sample was run concurrently with each assessment and all results were expressed as a percentage of this standard Levey–Jennings plots were made with this standard to ensure that performance complies with Westgard rules for acceptance of the experimental run.

**Figure 1:**
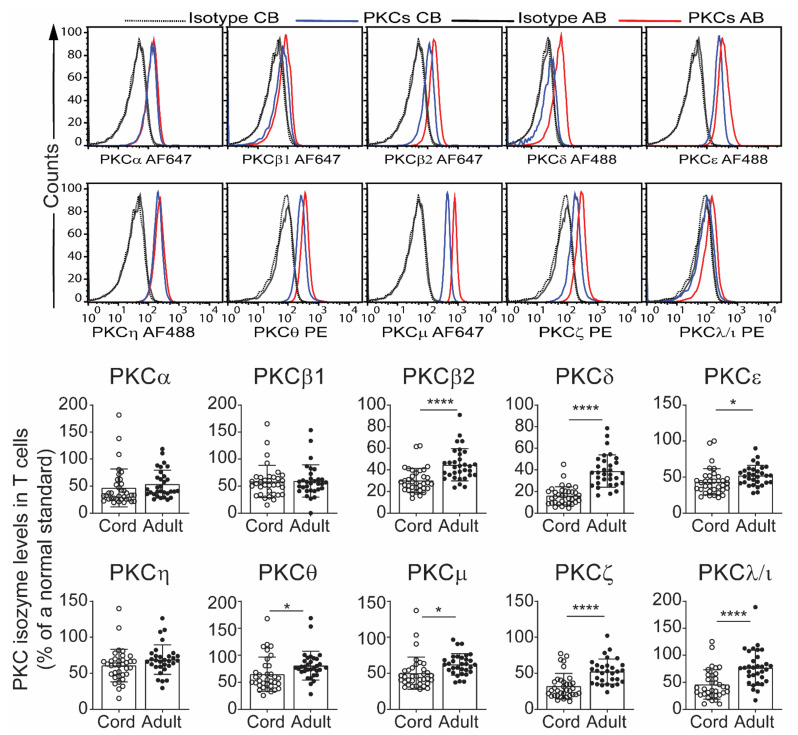
Protein kinase C (PKC) isozyme expression by CD3^+^ T cells from cord blood (CB) and adult blood (AB). Whole CB (*n* = 35) and adult blood samples (*n* = 31) were treated with fluorochrome labelled anti-CD3 monoclonal antibody, then cells were permeabilized for intracellular staining using fluorochrome tagged isozyme specific monoclonal antibodies. The cells were then analysed by flow cytometry. Supplementary Figure S1 shows the gating strategies. The median fluorescent intensity (MFI) (after deducting the isotype control values) was obtained and normalized against the standard cryopreserved T cells, run at the same time, and represented as a % of this standard. Data are presented as mean ± SD. * *p* < 0.05, **** *p* < 0.0001 (Student’s *t*-test). The top histogram panels show an example of a cord blood and an adult blood sample.

The data presented in Figure 1 show that CB CD3^+^ T cells express low levels of PKCβ2, δ, μ, ε, θ, ζ and λ/ι but not PKCα, β1 and η compared to T cells from adults (Figure 1). The results are expressed as both the individual values and mean ± SD of the indicated number of samples. The top panel shows an example of an individual sample run, by histograms and the gating strategy used is shown in Supplementary Figure S1.

### 2.2. PKC Isozyme Expression in CB CD4^+^ and CD8^+^ T Cells

**Figure 2:**
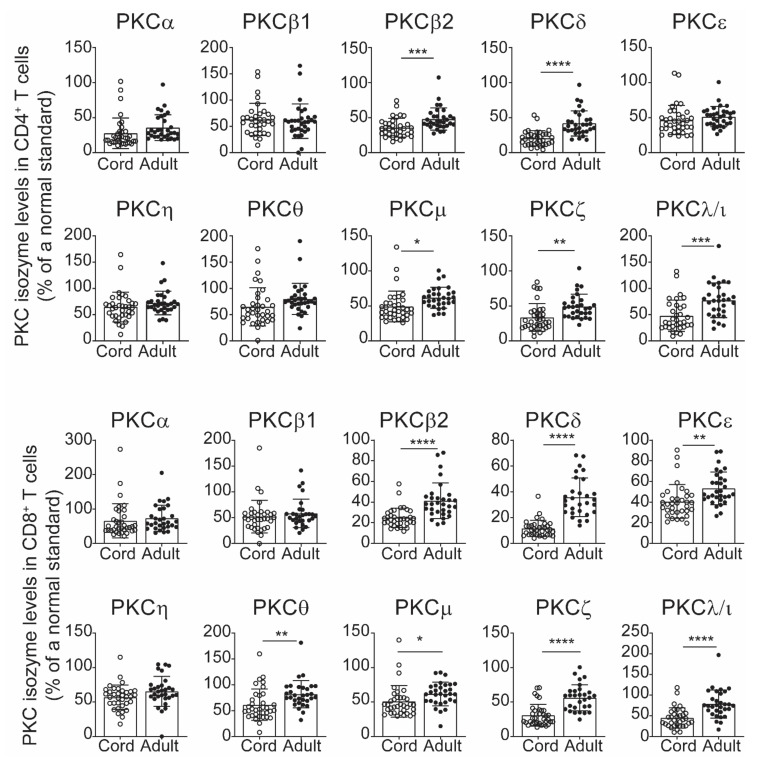
PKC isozyme expression in CD4^+^/CD8^+^ T cells from CB and AB. Whole blood from cord (*n* = 35) and adult (*n* = 31) were treated with fluorochrome labelled anti-CD3 and anti-CD8 monoclonal antibodies and then with fluorochrome tagged isozyme specific monoclonal antibodies for intracellular PKC isozyme detection. Data are expressed as per Figure 1 legend and presented as mean ± SD. * *p* < 0.05, ** *p* < 0.01, *** *p* < 0.001, **** *p* < 0.0001 (Student’s *t*-test).

**Figure 3:**
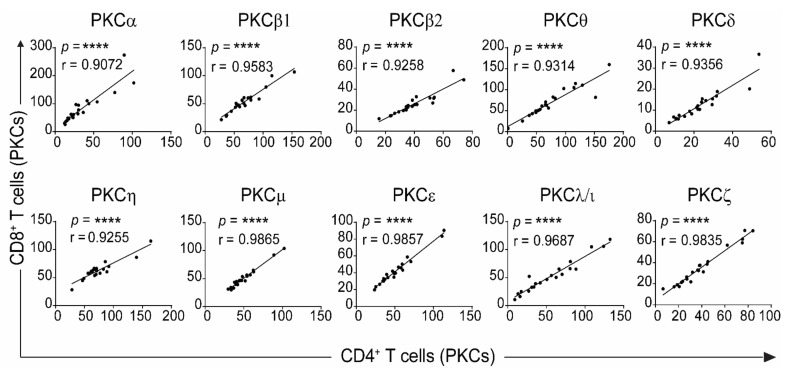
Correlation of levels of PKC isozyme expression between CD4^+^ and CD8^+^ CBTC. Data from Figure 2 were subjected to correlation analysis to deduce the relationship of PKC isozyme expression (MFI) between CD4^+^ and CD8^+^ T cells. **** *p* < 0.0001. Correlations were performed using the two-tailed Pearson correlation.

In all previous studies, the levels of PKC isozymes were measured in total T cells and not the T cell subpopulations. Here the levels in the CD4 and CD8 subsets were examined in the event that we could gain greater specificity by using the subset values. The same blood samples as in Figure 1 were analysed for PKC isozyme expression using the anti-CD3 and anti-CD8 monoclonal antibodies and PKC isozyme specific monoclonal antibodies. The data presented in Figure 2 show that CB CD4^+^ T cells express low levels of PKCβ2, δ, μ, ζ and λ/ι but not PKCα, β1, ε, η and θ compared to CD4^+^ T cells from adults. Examination in the CD8^+^ T cell subset showed that PKCβ2, δ, μ, ζ, ε, θ and λ/ι were low (Figure 2). This most likely accounts for the same isozymes being low in the total T cells. However, the data presented in Figure 3 show that there is a highly significant correlation in the PKC isozyme levels between the CD4 and CD8 subpopulations, indicating that the total T cell PKC isozyme levels are representative of both the CD4^+^ and CD8^+^ T cells.

### 2.3. Transient Nature of the PKC Isozyme Deficiencies in CBTC during Maturation

**Figure 4:**
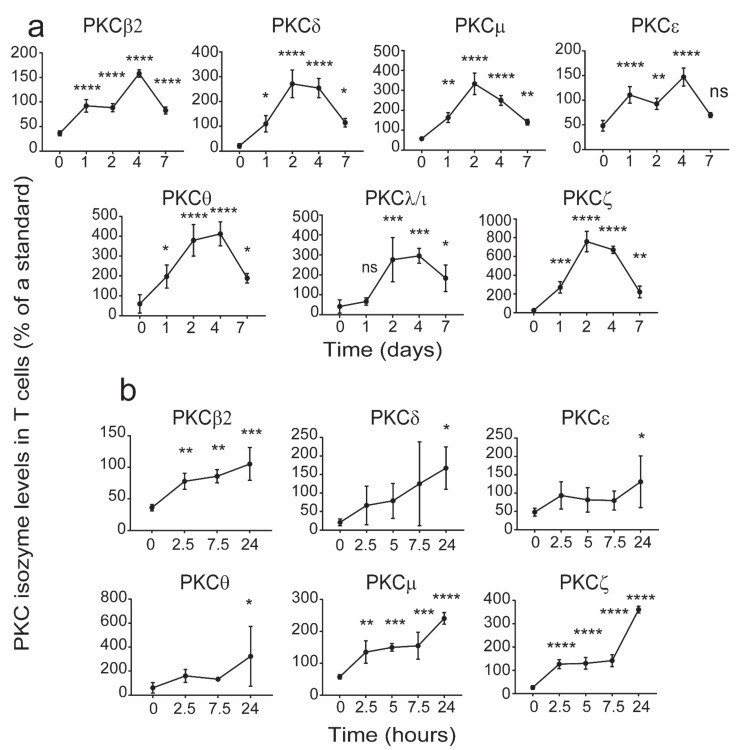
Changes in levels of CB CD3^+^ T cell PKC isozyme levels during 7-day maturation in culture. CBMCs containing T cells expressing low PKC isozymes levels were cultured in the presence of phytohaemagglutinin (PHA) (2 μg/mL) and rhIL-2 (10 ng/mL) and then the levels of PKC isozymes were determined over seven days by flow cytometry (**a**). For those isozymes which achieved significance at 1 day, the levels were examined prior to 24 h culture period (**b**). The MFI for the PKCs is expressed as a % of the control standard and as mean ± SD (*n* = 4 each with cells from a different individual). * *p* < 0.05, ** *p* < 0.01, *** *p* < 0.001, **** *p* < 0.0001 compared to day-0. ns: not significant. One-way ANOVA with *post hoc* Tukey’s multiple comparisons test.

**Figure 5:**
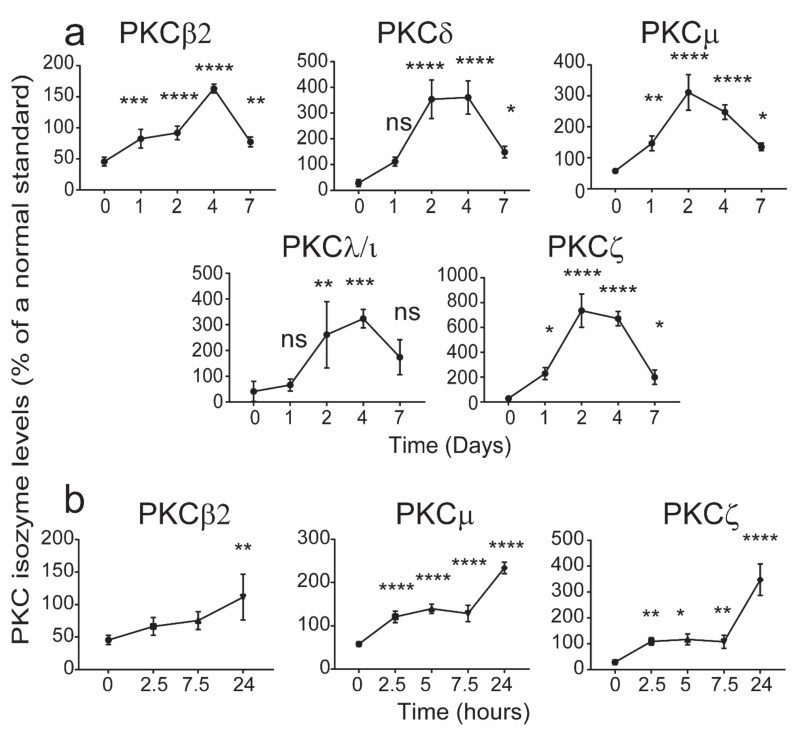
Changes in levels of CD4^+^ CBTC PKC isozymes levels during 7-day maturation. CBMCs with low PKC isozyme expressing PKC isozymes CD4^+^ T cells were cultured in the presence of PHA (2 μg/mL) and rhIL-2 (10 ng/mL) for 7 days. The levels of PKC isozymes were determined during this culture period (**a**). For those isotypes which achieved significance at 1 day the levels were re-examined prior to 24 h culture period (**b**), by flow cytometry. The MFI for the PKCs is expressed as a % of the control standard and as mean ± SD (*n* = 4 each with cells from a different individual). * *p* < 0.05, ** *p* < 0.01, *** *p* < 0.001, **** *p* < 0.0001 compared to day 0. ns: not significant. One-way ANOVA with *post hoc* Tukey’s multiple comparisons test.

**Figure 6:**
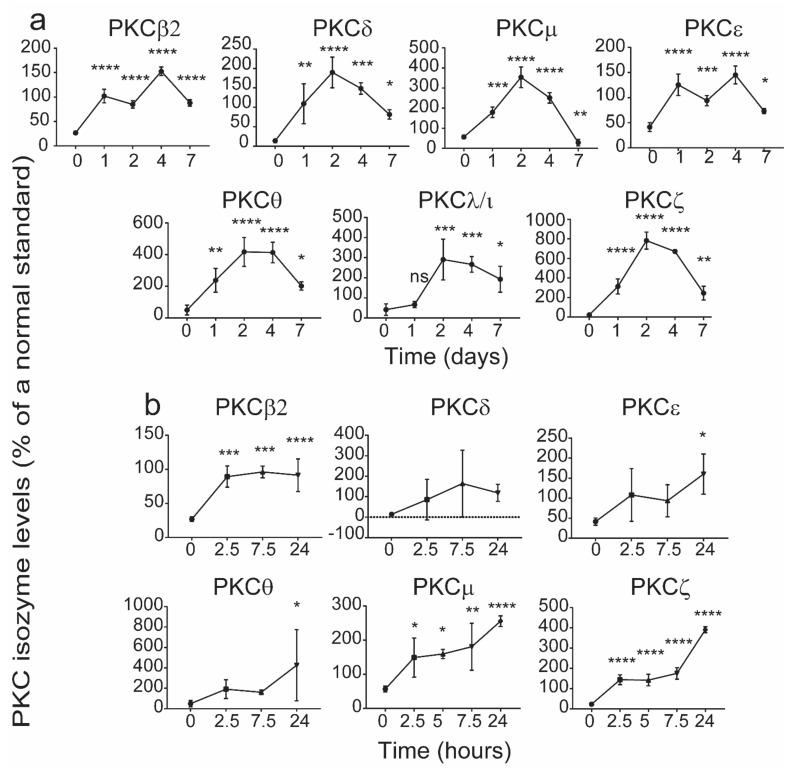
Changes in levels of CD8^+^ CBTC PKC during 7-day maturation. CBMCs with low PKC isozyme expressing CD4^+^ T cells were cultured in the presence of PHA (2 μg/mL) and rhIL-2 (10 ng/mL) for 7 days. The levels of PKC isozymes were determined during this culture period (**a**). For those isotypes which achieved significance at 1 day, the levels were re-examined prior to 24 h culture period (**b**), by flow cytometry. The MFI for the PKCs is expressed as a % of the control standard and as mean ± SD (*n* = 4 each with cells from a different individual). * *p* < 0.05, ** *p* < 0.01, *** *p* < 0.001, **** *p* < 0.0001 compared to day-0. ns: not significant. One-way ANOVA with *post hoc* Tukey’s multiple comparisons test.

In order to determine this window, the changes in the levels of each of the PKC isozymes were determined in an in vitro culture model of CB mononuclear cells (CBMCs) treated with phytohaemagglutinin (PHA). CB samples in which the T cell PKC isozyme levels were less than the 5th percentile [14] were used. In these cultures, recombinant human interleukin-2 (rhIL-2) was added on the 3rd day after initiating the culture to ensure cell survival during maturation. At the times indicated in Figure 4, the cells were harvested and examined for levels of PKC isozymes by flow cytometry. The data presented in Figure 4 show that T cell PKC isozyme expression increased to within the 5th and 95th percentiles [12] of adult levels by day 7. As the levels were normalized (seen after maturation) within 24 h of culture for some of the isozymes, we attempted to identify the levels of PKC isozymes prior to this time. The levels of PKCζ and μ in the T cells had normalised by 2.5 h (Figure 4), while other PKC isozymes took at least 24 h to show a significant increase (Figure 4). It is noted that this was also the case when examining the CD4^+^ and C8^+^ T cell subsets (Figure 5 and Figure 6). The data identify a period during which levels of the deficient PKC isozymes are increased to within the normal adult range.

### 2.4. Relationship between CBTC PKC Isozyme Levels and Cytokine Production in the Matured Cells

Previous studies showed that the levels of PKCζ at birth were associated with the development of T cells correlating positively with IFN-γ production and negatively with IL-9 [14]. Here we examined whether there is any correlation between the levels of expression of other PKC isozymes in CB CD3^+^ T cells and cytokines produced following their maturation. Purified CBTCs were matured in culture by using anti-CD3/-CD28 antibodies and rhIL-2. On day 7, the matured cells (Figures S2 and S3 show the proportions of naïve and memory markers expression in T cells and T cell subsets at day 7) were then harvested and stimulated with PHA and Phorbol 12-myristate 13-acetate (PMA). The number of cytokine-producing cells were measured by flow cytometry. The matured cells produced IFN-γ, IL-2, LT-α, TNF, IL-4, IL-5, IL-9, IL-10, IL-I3, IL-17, IL-21, IL-22 and TGF-β [14] (Figure S4).

Examination of the relationship between the PKC isozyme levels in CBTC and cytokine patterns produced by the matured T cells showed that apart from PKCζ there was essentially no correlation with the other PKC isozymes (Table 1). Analysis revealed that PKCζ correlates positively with Th1 cell cytokines IFN-γ, IL-2 and TNF and negatively with a subset of Th2, Th9 cell type (IL-9) development and IL-10 production in CD3^+^ cells (Table 1). This was observed both as analysis of % cytokine-positive cells and the MFI. While there was some correlation between PKCδ and IFN-γ/IL-4, both cytokines showed a positive correlation (Table 1).

Assessing these cytokine patterns for correlation with PKC isozyme levels in CB CD4^+^ and CD8^+^ T cells showed a similar correlation to the CD3^+^ T cells (Supplementary Figures S5 and S6). In some cases, while cytokine correlation was not significant in terms of % T cells, a correlation was also observed when assessed against the cytokine level (MFI levels).

## 3. Discussion

The current data on naïve CBTC PKC isozyme expression show reduced levels of PKC isozymes in CB CD3^+^ T cells compared to levels in peripheral blood CD3^+^ T cells from adults. The expression levels of PKCβ2, δ, μ, ζ, ε, θ and λ/ι isozymes in CB CD3^+^ T cells were significantly reduced and the data extend the previous findings on reduced levels of PKC isozymes in CBTC, based on Western blot assays [10]. In the main, findings showed that the isozyme deficiency between the first [8], second [13] and this study was in agreement. However there was a difference in either β1 or θ expression, perhaps due to the cohorts studied; while the initial studies were from *ad hoc* deliveries [10], the other involved women with a history of allergic diseases [8] and the present study involved caesarean births. While there was a difference between each of the two published reports [8,10] and this study, with respect to either β1 or θ, this could be explained in the cohorts where in initial studies it was not selective [10], but in the other it involved those with a history of allergic diseases [8], and in the present study they were from caesarean births. With low PKCζ levels in CBTCs known to be correlated with increased risk of allergy and the maturation of the T cells towards a Th2 bias [14], we examined whether the expression of the other PKC isozymes correlated with the expression of specific cytokines patterns. Only PKCζ has a positive association with T cell maturation towards a Th1 cell cytokine bias (e.g., IFN-γ) and a negative association with Th9 (IL-9) cell development, with no associations between the other deficient PKC isozymes and specific cytokine patterns. These findings support previous observations that only CBTC PKCζ levels correlated with allergy sensitization and the risk of developing allergy in childhood [8,9,15]. Furthermore, knocking down of PKCζ expression in CBTC skewed their development towards a Th2 cytokine phenotype [5].

Examination of the CB CD4^+^ and CD8^+^ T cells revealed that essentially similar PKC isozymes were deficient in the subpopulations as in the total T cells with some differences in PKCε and θ for the CD4^+^ T cells. Indeed, the PKC isozyme expression between CD4 and CD8 cells was significantly correlated. This suggests that measurements in the total T cell population as a biomarker are appropriate. While CD8 T cells are known for their cytotoxic function, e.g., against either virus-infected cells, cancer cells, or both [16], as well as immunosuppressive actions [17], there is increasing evidence suggesting their critical role in allergy development [18,19]. Asthmatic patients with reduced number of IFN-γ^+^ CD8 T cells (Tc1) and high frequency of IL-13^+^ CD8 T cells (Tc2) was associated with severity of disease [20]. High frequency of IL-9^+^ CD8 T cells (Tc9) have been associated with eosinophilia and high fractioned exhaled nitric oxide of allergic asthma [21]. Thus, it is not surprising that the associations with PKCζ are similar for CD4 and CD8 T cells.

A control on allergy may be achieved by regulating cytokine production, which, based on our previous [5,8,9,14] and present findings, both are linked to the expression of PKCζ. CD4^+^ Th2 cytokines, such as IL-4 (class switch to IgE in B cells), IL-5 (development, survival, activation and differentiation of eosinophils, mast cells and enhanced degranulation of basophils) and IL-13 (class switch to IgE, activation of mast cells, enhanced epithelial cell mucus production and augmented eosinophil trafficking to mucosal sites), are the hallmark of allergic conditions [11,22,23,24]. IL-9 is produced by Th9 cells which are a subset of Th2 cells expressing peroxisome proliferator-activated receptor gamma [25], which develop in the presence of transforming growth factor-beta (TGF-β) and IL-4 [26,27,28]; IL-9 plays a role in allergy development [29,30,31,32,33] by increasing IgE expression on B cells [34] and causing bronchial hyperresponsiveness in inhaled methacholine-induced asthma [14,35]. IL-9 also plays a pathogenic role in allergic asthma by controlling mast cells (survival and proliferation, increases expression of proteases and FcεRI expression [32,36,37,38]), epithelial cells (increase mucus production) and promotes airway eosinophilia (eosinophil survival, differentiation and IL-5 Receptor expression [39,40]). The Th1 cytokine, IFN-γ, inhibits Th2 and Th9 development and survival [22,23,24,26].

The low expression of PKC isozymes in CBTC appears to be a transient state that may be critical in determining the fate of T cell maturation following exposure to different environments. However, the ‘normalisation’ of these isozymes is required to elicit effective immune responses, once matured, irrespective of whether there is a propensity towards a Th1 or Th2/Th9 response. Here we demonstrate that the PKC isozymes that are expressed in deficient amounts in CBTC increase following the addition of the maturation signal, PHA. This occurred rapidly within the first 24 h of culture suggesting that the window of opportunity to modify these levels for intervention purposes is short. Previously we have demonstrated that supplementing women during pregnancy with n-3 polyunsaturated fatty acids (fish oil), results in significant increases in PKCζ levels in CBTC from their babies [8,15]. This increase was likely to be epigenetically controlled through the acetylation of histone H3 in the *PKCZ* gene promoter region [5]. Given this response to n-3 fatty acid supplementation, it is possible that PKCζ levels could also be altered by using other environmental approaches, provided that these manipulations occurred prenatally or early perinatally. Although the increased prevalence of allergies worldwide is widely recognised, the reason behind this is still unclear. A rather stable population genetic profile could not explain the increased prevalence of atopy and allergy in industrialized countries, but a skewed exposure toward harmful rather than protective epigenetically-driven environmental factors is more plausible [41], which may act at this period of low PKCζ. This now provides a basis for further exploration of the characteristics of the deficiency in neonates as a prelude to attempt interventions to decrease the risk of allergy development.

As PKCζ has been found to be a principal biomarker that can predict allergy development risk [8,9], this enzyme can potentially be targeted to prevent allergy development, by raising its levels in utero [8]. For the first time, the current findings provide evidence of the existence of a time window during the postnatal period when environmental influences could be applied to achieve epigenetic modulation of PKCζ, such as through a simple low-risk nutritional strategy employing fish oil supplementation. Future studies should be aimed to characterize this transient deficiency in neonates during maturation in vivo.

## 4. Materials and Methods

### 4.1. Reagents

Cell culture reagents used include RPMI 1640 medium supplemented with L-Glutamine (Thermo Fisher Scientific, Waltham, MC, USA, 11875-093), X-VIVO 15 medium (Lonza, BSL, Switzerland, 04418Q) and foetal calf serum (FCS) (Serana, Pessin, German, FBS-AU-015). PHA, PMA and human AB serum were obtained from Sigma Aldrich (St. Louis, MO, USA). The rhIL-2 was purchased from PeproTech (Rocky Hill, NJ, USA). The monoclonal antibodies against PKC isozymes and cytokines are listed in Table 1, Table 2 and Table 3 with all the relevant details.

### 4.2. Ethics Statement

Human blood sample collection and all experimental procedures were approved by the Human Research Ethics Committee of the Women’s and Children’s Health Network (WCHN), Adelaide, South Australia, in accordance with the National Statement on Ethical Conduct in Human Research (2007, updated 2018) (National Health and Medical Research Council Act 1992). Venous blood was collected from adult volunteers who normally donated blood as ‘healthy’ controls for pathology testing, with their informed consent. The gender of the donors was 15 females and 16 males. Umbilical cord blood (CB) from healthy neonates was obtained with informed consent from pregnant women undergoing elective caesarean section. Gestation age was from 37 to 39 weeks (with 28 of the 35 being 38–39 weeks), and only one of these babies also had a sibling involved. Of the 35 babies, 17 were females and 18 were males.

### 4.3. Preparation of Mononuclear Cells (MC) from CB and Peripheral Blood of Adult Donors

CB and adult blood in lithium-heparin were applied to Ficoll Paque Plus media (GE Healthcare, Uppsala, Sweden) density gradient centrifugation and the MC which resolved at the interface harvested. The cells were washed in complete medium (RPMI-1640 supplemented with L-glutamine (100 U/mL), penicillin (100 μg/mL), streptomycin (100 μg/mL) and 10% FCS). The cells were cryopreserved at 5–10 × 10^6^/mL in cell freezing media containing FCS (90%) and DMSO (10%) as previously described [12].

### 4.4. Isolation of T Cells

The T cells were isolated by negative selection using the EasySep Human CD3^+^ T cell Isolation Kit (Stemcell Technologies, Vancouver, BC, Canada). The MCs were rapidly thawed and washed in separation buffer (phosphate-buffered saline (PBS) containing 2% FCS and 1 mM EDTA). Viability assessed by Trypan blue exclusion was approximately 90%. The cells at 5 × 10^7^ cells/mL of separation buffer, in polystyrene round-bottom tubes (Corning, AZ, USA, 352058) were incubated with 50 μL/mL of the EasySep Human CD3^+^ T Cell Isolation Cocktail for 5 min. Furthermore, 50 μL/mL of EasySep Dextran Rapid Spheres was added to cells and the non T cells were removed with an EasySep Magnet (Stemcell Technologies, Vancouver, BC, Canada, 18000). The resultant CBTCs preparation had a purity of ≥97%.

### 4.5. CBTC Maturation

CBTCs were matured using an established model. The cells were cultured in the presence of anti-CD3 and anti-CD28 antibodies [14]. Briefly, 2.5 μg/mL anti-CD3 antibodies (OKT3, Abcam, Cambridge, UK) were immobilised in 24 well plates in Hank’s balanced salt solution (HBSS). The plates were refrigerated overnight or incubated for 3 h at 37 °C, and then washed with HBSS. At the initiation of the culture, to 1 × 10^6^ CBTC was added 1 µg/mL anti-CD28 antibodies (Clone CD28.2, eBiosciences, San Diego, CA, USA) in a total volume of 1 mL. In some assays, CBMC at 1 × 10^6^ cells/mL in complete media were matured by using PHA (2 µg/mL, final concentration). On the 3rd day, the cells were counted and reseeded at 1 × 10^6^/mL with rhIL-2 (10 ng/mL). This was repeated on day 5. The expression of CD45RA/RO surface expression was measured on day 7 by flow cytometry. The cells were used for cytokine production studies as required.

### 4.6. Flow Cytometric Detection of PKC Isozymes

The expression of PKC isozymes was assessed as described previously [12]. Briefly, either whole blood or 2 × 10^5^ CBMCs were incubated with anti-CD3 APC-H7 and anti-CD8 PE-Cy7, both from BD Biosciences (Franklin Lakes, NJ, USA) for surface staining for 15–20 min. The cells were then fixed with BD Cytofix/Cytoperm (BD, 555028) and permeabilized with NET-Gel. Furthermore, 1 µg of mouse/rabbit IgG Fc blocking reagent was added for 10 min. The optimal amount of fluorochrome-conjugated anti-PKC isozymes antibodies or isotype controls (Table 2) were added as appropriate. After 30 min of incubation at room temperature (RT) in the dark, the cells were washed twice. They were then analysed on a FACSCanto II (BD Biosciences, NJ, USA).

### 4.7. Measurement of Intracellular Cytokines

The assays were conducted essentially as previously described using BD Cytofix/Cytoperm Plus Permeabilization Kit with GolgiPlug [14]. Briefly, the T cells at 1 × 10^6^/mL in RPMI-1640/2.5% AB serum were stimulated with PMA (50 nM) and PHA (2 μg/mL) in the presence of Brefeldin A (GolgiPlug) and incubated at 37 °C/5% CO_2_ for 16–20 h. Cells were washed and incubated with BD Horizon Fixable Viability Stain 510 (FVS510) stock solution (1:1000) in protein- and sodium azide-free PBS for 15 min. After washing, cells were treated with anti-CD45 APC-H7 (2D1) and anti-CD3 PE-Cy5 (HIT3a) and incubated at RT in the dark for 15–20 min. The cells were fixed with BD Cytofix/CytoPerm solution for 20 min and permeabilized with BD Perm/Wash solution for 10 min at RT in the dark. For detection of intracellular cytokine, cells were treated with fluorochrome-conjugated antibodies (see Table 3 and Table 4) and then incubated for 30 min at RT in the dark. After the incubation, cells were washed twice with BD Perm/Wash; the samples were analysed on a FACSCanto II. Data analysis was performed using FlowJo v10.1 for cytokine expressing T cells. Cells were analysed after the exclusion of doublets and dead cells by gating on the FVS510^−^ cell population.

### 4.8. Statistical Analysis

Statistical analyses were conducted by one-way ANOVA followed by *post hoc* Tukey’s multiple comparisons test, or Student’s *t*-test. The two-tailed Pearson correlation coefficient was used for examining data for the presence of correlation. These were performed using GraphPad Prism v9 (GraphPad Software, La Jolla, CA, USA). A *p* value of < 0.05 was considered significant.

## Figures and Tables

**Table 1:** Correlation analysis of PKC isozymes and cytokine production in matured CD3^+^ cells ^#^.

Cytokines	PKCα	PKCβ1	PKCβ2	PKCδ	PKCε	PKCη	PKCθ	PKCμ	PKCζ	PKCλ/ι
IFN-γ (%)	0.5604	0.1782	0.4377	0.7085 *	0.4995	0.5377	0.1967	0.3937	0.7019 *	0.0939
IFN-γ (MFI)	0.2106	0.1976	0.1796	0.3579	0.3686	0.1659	0.5463	−0.0134	0.6768 *	−0.1691
IL-2 (%)	0.2204	0.3329	0.1008	0.3092	0.2727	0.1757	0.3761	−0.0376	0.5745	−0.2421
IL-2 (MFI)	0.2446	0.1459	0.2505	0.4598	0.2192	0.2446	0.4263	0.2310	0.7079 *	−0.0340
TNF (%)	0.3748	0.2478	0.2076	0.4679	0.3332	0.2666	−0.0105	0.0986	0.5599	−0.0989
TNF (MFI)	0.3008	0.3182	0.1975	0.4791	0.3996	0.2009	0.3994	0.0351	0.7659 **	−0.1134
LT-α (%)	0.3346	0.0347	0.2112	0.3929	0.3334	0.1884	−0.2018	0.1209	0.3434	0.0537
LT-α (MFI)	−0.0937	0.0765	−0.2068	−0.0380	−0.0044	−0.1691	−0.2348	−0.3628	0.2329	−0.4143
IL-4 (%)	0.4928	0.2634	0.2714	0.6645 *	0.4175	0.3514	−0.0338	0.2853	0.5840	0.0918
IL-4 (MFI)	0.2515	0.2140	0.0579	0.4423	0.1989	0.0969	−0.1759	0.0935	0.4623	−0.1301
IL-5 (%)	0.2875	0.5194	0.1459	0.0834	0.5722	0.1974	0.4155	−0.2627	0.2405	0.0372
IL-5 (MFI)	−0.0224	0.1130	−0.1156	−0.0436	0.3052	−0.1455	0.1579	−0.3850	0.0816	−0.1837
IL-9 (%)	−0.4542	−0.4489	−0.3473	−0.6409 *	−0.4878	−0.3095	−0.4324	−0.1984	−0.8345 **	−0.03258
IL-9 (MFI)	−0.5337	−0.6502	−0.4375	−0.6236	−0.5397	−0.4115	−0.4924	−0.1557	−0.881 ***	−0.1508
IL-10 (%)	−0.2731	−0.3727	−0.1408	−0.4348	−0.2676	−0.0802	−0.0477	−0.0500	−0.6230	0.1204
IL-10 (MFI)	−0.4495	−0.3071	−0.2806	−0.5754	−0.4010	−0.3320	−0.1614	−0.2134	−0.6725 *	0.0095
IL-13 (%)	−0.1708	−0.1348	−0.1926	−0.1691	−0.0013	−0.0055	0.4277	−0.1217	−0.2527	−0.1113
IL-13 (MFI)	−0.3522	−0.3389	−0.3298	−0.4816	−0.3356	−0.3757	0.0836	−0.2117	−0.5320	−0.0616
IL-17 (%)	−0.083	−0.5377	0.0448	−0.2234	−0.0655	0.1499	0.0774	0.1560	−0.5995	0.2036
IL-17 (MFI)	0.2200	0.6979 *	0.1645	−0.0019	0.3411	0.0338	0.1120	−0.3631	0.4925	−0.0400
L-21 (%)	0.5002	0.3347	0.2669	0.4598	0.5308	0.3461	−0.0795	0.1010	0.5185	0.1458
IL-21 (MFI)	0.0205	−0.2084	−0.0434	0.04250	−0.1240	−0.0438	−0.3547	0.1681	0.0046	−0.0509
IL-22 (%)	0.0561	−0.0125	0.0720	0.2144	0.2900	0.08116	0.7090 *	−0.03731	0.4114	−0.2888
IL-22 (MFI)	0.2119	0.4719	−0.0041	0.3309	0.3908	0.04281	0.0788	−0.2496	0.4745	−0.2407
TGF-β (%)	0.2417	−0.6425	0.3993	0.1699	0.2979	0.5000	0.5319	0.5087	−0.291	0.3904
TGF-β (MFI)	0.1783	−0.0354	0.2476	−0.0299	0.4619	0.1653	0.5937	−0.1791	0.3515	0.0906

^#^ Data represents Pearson correlation (r). Samples *n* = 10. * *p* < 0.05, ** *p* < 0.01, *** *p* < 0.001. Correlations were performed using the two-tailed Pearson correlation.

**Table 2:** Staining panel for the determination of PKC isozymes expression.

Antibody (Clone)	Fluorochrome	Cat/Company
Anti-PKCα (H-7)	AF647	sc-8393/Santa Cruz
Anti-PKCβII (F-7)	AF647	sc-13149/Santa Cruz
Anti-PKCβ1 (EPR18512)	AF647	ab223452/Abcam
Anti-PKCζ (H-1)	PE	sc-17781/Santa Cruz
Anti-PKCθ (E-7)	PE	sc-1680/Santa Cruz
Anti-PKCλ/ι (H-12)	PE	sc-17837/Santa Cruz
Anti-PKCη (EPR18513)	AF488	ab179524/Abcam
Anti-PKCδ (EPR17075)	AF488	ab206282/Abcam
Anti-PKCε (EPR1482)	AF488	ab217980/Abcam
Anti-PKCμ (EP1493Y)	AF647	ab51246/Abcam
Mouse IgG1k Isotype control (MOPC-31C)	AF647	566011/BD
Rabbit mAb IgG Isotype Control	AF488	2975/CST
Rabbit mAb IgG Isotype Control	AF647	2985/CST
Mouse mAb IgG2ak (X39)	PE	340459/BD
Anti-CD3 (SK7)	APC-H7	560176/BD
Anti-CD8 (RPA-T8)	PE-Cy7	557746/BD

BD—BD Biosciences (Franklin Lakes, NJ, USA), CST—Cell Signaling Technologies (Danvers, MA, USA), Santa Cruz—Santa Cruz Biotechnology (Dallas, TX, USA), Abcam—Abcam (Cambridge, UK).

**Table 3:** Antibodies panel#1 for T cells cytokines.

Antibody (Clone)	Fluorochrome	Cat/Company
Anti-IL-2 (5344.111)	BV421	562914/BD
Anti-IL-10 (JES3-9D7)	AF488	501413/BioLegend
Anti-LT-α (359-81-11)	PE	554556/BD
Anti-IL-17A (N49-653)	PerCP-Cy 5.5	560799/BD
Anti-TGF-βI (TW4-2F8)	PE/Cy7	349610/BioLegend
Anti-TNF (MAb11)	APC	554514/BD
Anti-IFN-γ (4S.B3)	APC/Cy7	502530/BioLegend
	BV510 (viability stain)	564406/BD

BD—BD Biosciences (Franklin Lakes, NJ, USA), BioLegend—BioLegend (SD, California, UK).

**Table 4:** Antibodies panel#2 for T cells cytokines.

Antibody (Clone)	Fluorochrome	Cat/Company
Anti-IL-13 (JES10-5A2)	BV421	563580/BD
Anti-IL-4 (MP4-25D2)	FITC	562047/BD
Anti-IL-21 (3A3-N2.1)	PE	562042/BD
Anti-IL-9 (MH9A3)	PerCP-Cy 5.5	561461/BD
Anti-IL-5 (TRFK5)	APC	562048/BD
Anti-IL-22 (2G12A41)	PE/Cy7	366707/BioLegend
Anti-IFN-γ (4S.B3)	APC/Cy7	502530/BioLegend
	BV510 (viability stain)	564406/BD

BD—BD Biosciences (Franklin Lakes, NJ, USA), BioLegend—BioLegend (SD, California, UK).

## Data Availability

The datasets generated during and analysed during the current study are available from the corresponding author on reasonable request.

## Supplementary Materials

The following are available online at https://www.mdpi.com/article/10.3390/ijms222312650/s1.
