# Supplementary material for: Characterization of the Transient Deficiency of PKC Isozyme Levels in Immature Cord Blood T Cells and Its Connection to Anti-Allergic Cytokine Profiles of the Matured Cells
Source: Int J Mol Sci. 2021 Nov 23;22(23):12650. doi: 10.3390/ijms222312650 (PMC8657888; doi:10.3390/ijms222312650)
Supplement: Supplementary file 1 [file ijms-22-12650-s001.zip › ijms-1446261-supplementary.PDF]

International Journal of *Molecular Sciences*

MDPI

2021-09-30

Article

Characterization of the Transient Deficiency of PKC Isozyme Levels in Immature Cord Blood T Cells and Its Connection to Anti-Allergic Cytokine Profiles of the Matured Cells

Khalida Perveen [1,2], Alex Quach [1,2], Michael J. Stark [2,3], Susan L. Prescott [4,5], Simon C. Barry [2], Charles S. Hii [1,2] and Antonio Ferrante [1,2,6,*]

* Correspondence: Antonio.Ferrante@adelaide.edu.au; Tel.: +61-8-81-617-216

Supplementary data

This file includes:

Figures S1 to S6

Tables S1 & S2

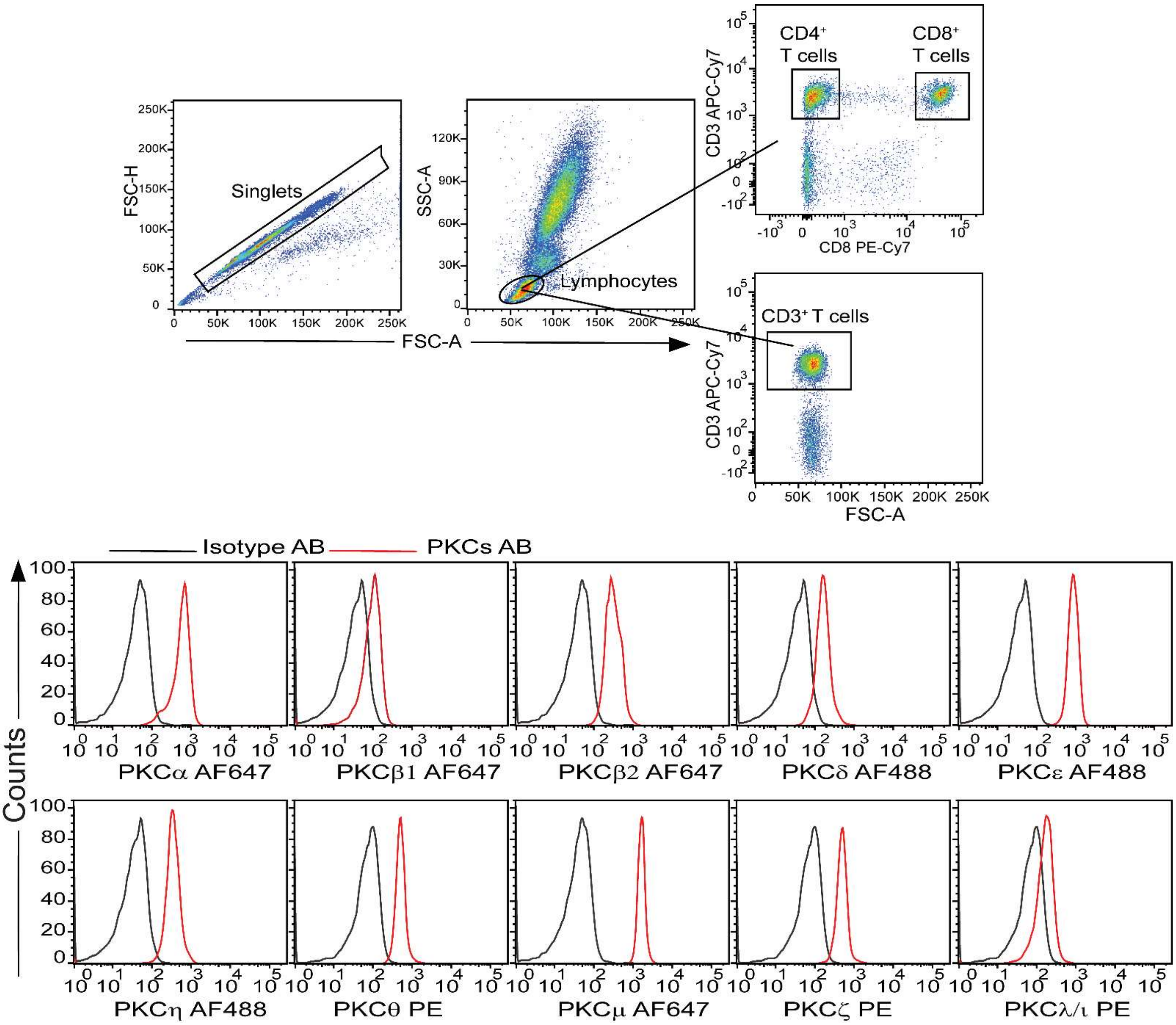

Figure S1. Gating strategies for T cell subsets in whole blood assays for PKC isozymes detection. Following surface and intracellular staining, which included red cell lysis, leukocyte fixation and permeabilisation; cells were acquired on the flow cytometer. Singlets were gated by FSC-A/FSC-H, and SSC was used to distinguish lymphocytes. The lymphocytes were further gated for CD3 or CD8 expression and further analysed for PKC isozymes expression by using the PKC antibody panel (Table 1). $CD4^+$ T cells were deduced by $CD3^+/CD8^-$ gating. Representative histograms are shown for PKC isozymes in $CD3^+$ T cells. The analysis is shown for whole adult blood.

International Journal of
Molecular Sciences

MDPI

Table S1. Antibodies panel for staining of Naïve/memory subsets in human CBTC.

**Antibody**	**Fluorochrome**	**Clone**	**Catalogue**	**Company**
Anti-CD45RA	FITC	HI100	555488	BD
Anti-CD45RO	PE	UCHL1	555493	BD
Anti-CD3	PE-Cy5	HIT3a	555341	BD
Anti-CD4	APC	RPA-T4	555349	BD
Anti-CD8	PE-Cy7	RPA-T8	557746	BD
Anti-CD45	APC-H7	2D1	641399	BD

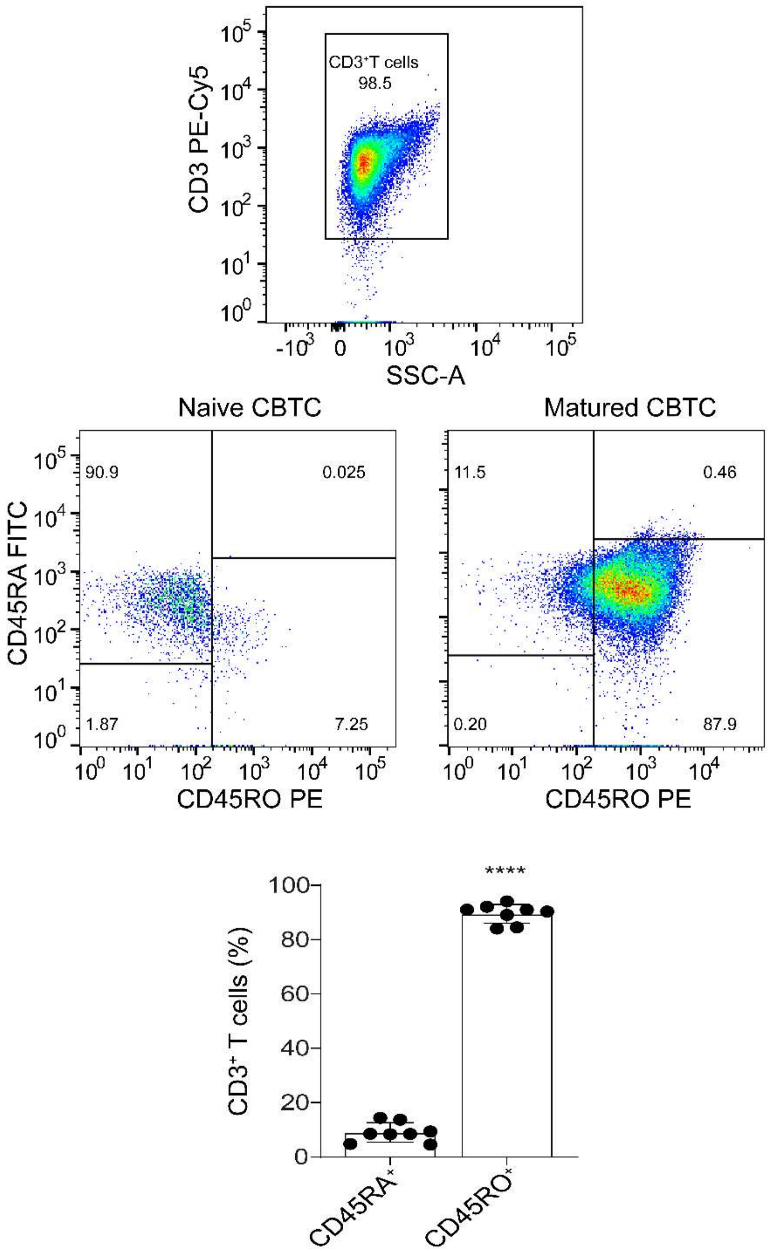

Figure S2. Proportions of naïve and memory T cells on day 7 of maturation. Purified CB CD3+ T cells were matured with anti-CD3/-CD28 and recombinant human interleukin-2 (rhIL-2) for 7 days. Then expression of CD45RA/RO was assessed by flow cytomentry. Representative dot plot for naïve and maturation expression in CB CD3+ T cells. The top panels show the dot plots of CD45RA/RO expression in T cells and the middle panels shows CD45RA/RO expression in naïve (left dot plot) and mature (right dot plot) in all the CBTC samples. Bottom panel shows data expressed as mean ± SD of n = 8 of expression on day 7. **** $p < 0.0001$. (Student's t-test).

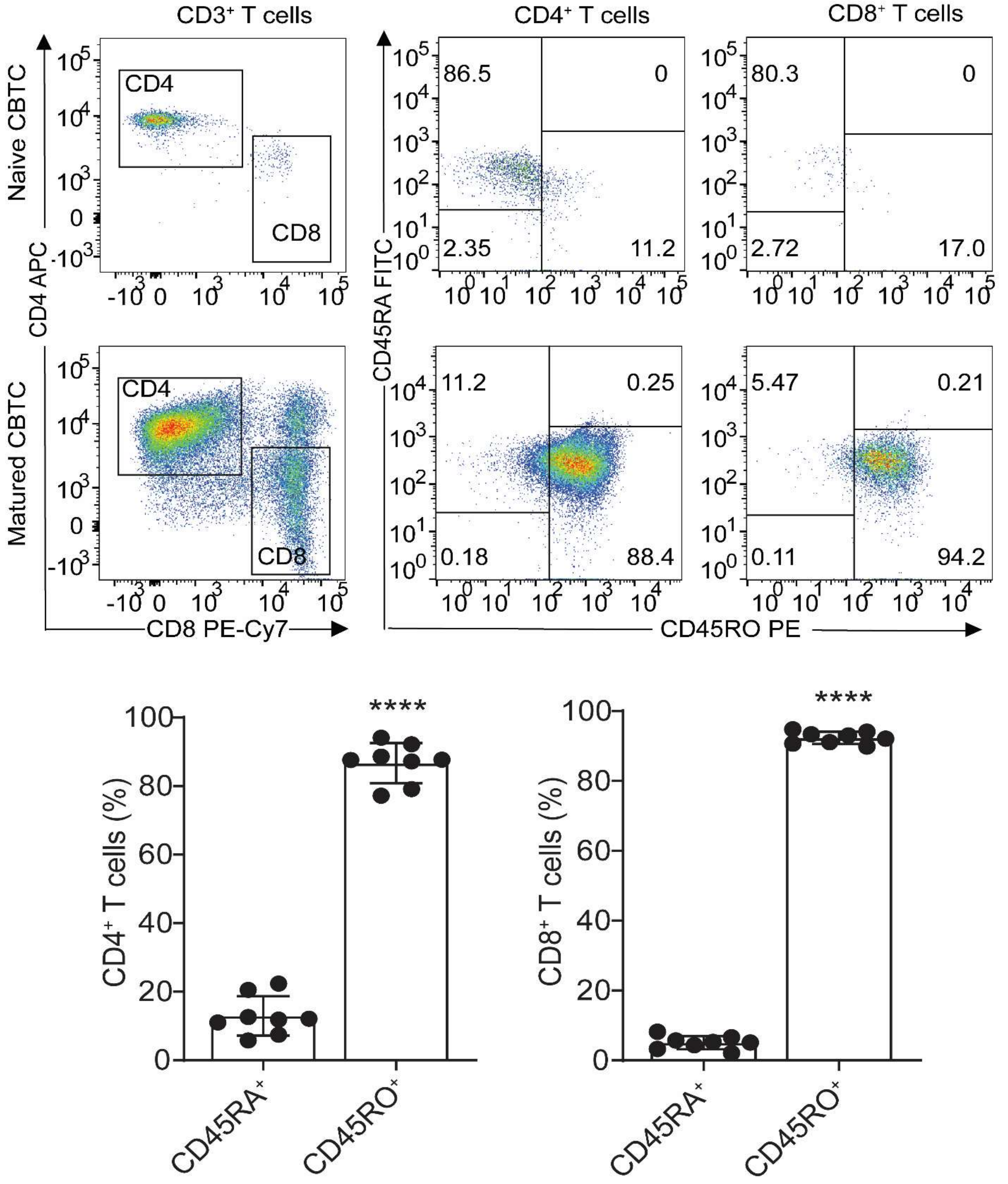

Figure S3. Proportions of naïve and memory CD4+/CD8+ T cells on day 7 of maturation. Purified CB CD3+ T cells were matured as in Figure S2. Representative dot plot for naïve and maturation expression in CB CD4+/CD8+ T cells. Right dot plots for gating CD4+/CD8+ T cells from total T cells. The CD45RA/RO expression in naïve (top dot plot) and mature (middle dot plot) in all the CB CD4+/CD8+ T cells samples. Bottom panels show data expressed as mean ± SD of n = 8. **** $p < 0.0001$. (Student's t-test).

International Journal of *Molecular Sciences*

MDPI

Table S2. Antibodies for detecting interleukin-4 (IL-4)-and interferon gamma (IFN-γ)-expressing T cells and CD4+/CD8+ T subsets.

Antibody	Fluorochrome	Clone	Catalogue	Company
Anti-IFN-γ	FITC	4S.B3	554551	BD
Anti-IL-4	PE	8D4-8	12-7049-42	eBioscience
Anti-CD3	PE-Cy5	HIT3a	555341	BD
Anti-CD8	PE-Cy7	RPA-T8	557746	BD
Anti-CD45	APC-H7	2D1	641399	BD
Mouse-IgG1k	FITC	MOPC-21	555748	BD
Mouse-IgG1k	PE	MOPC-21	556650	BD

International Journal of
Molecular Sciences

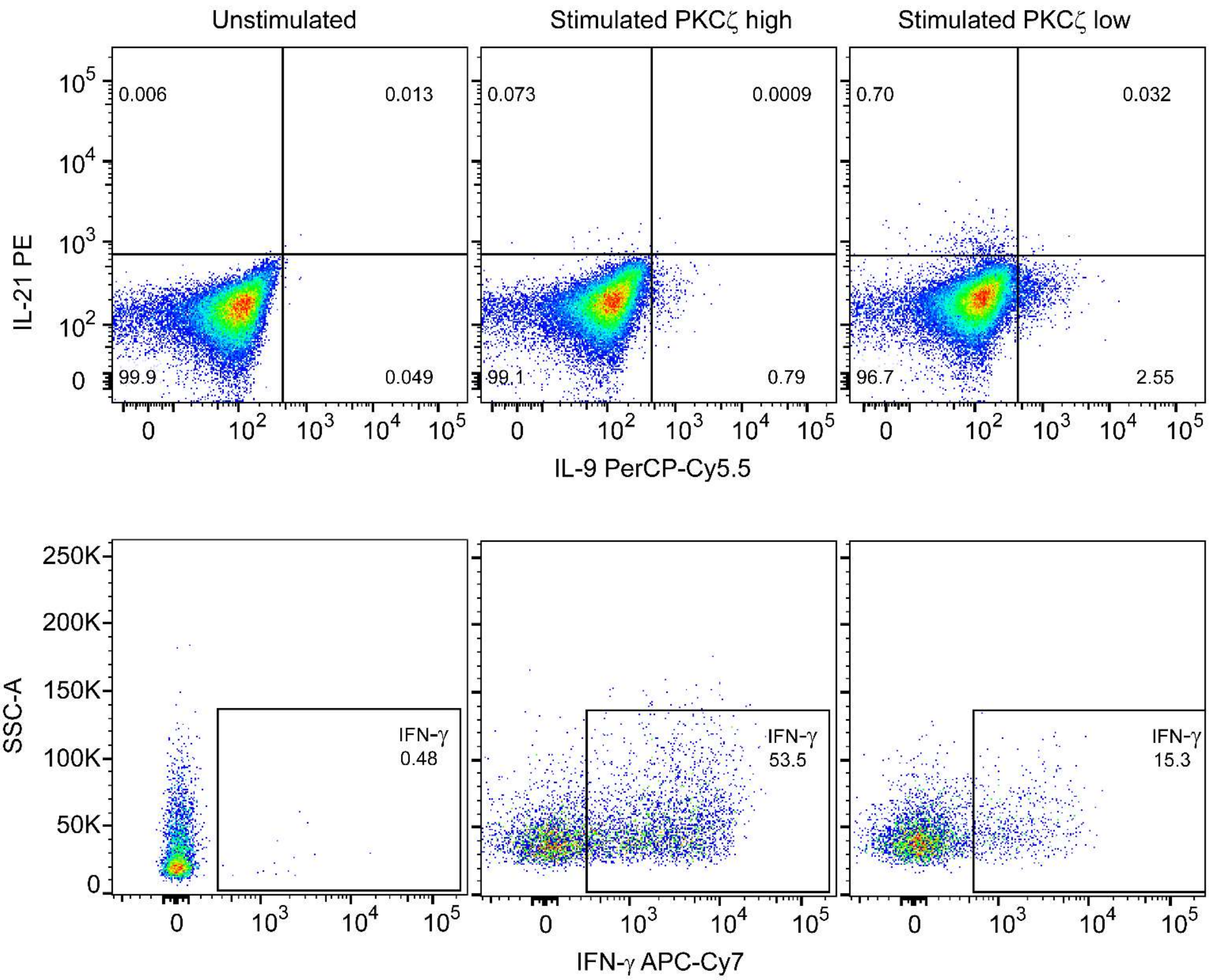

Figure S4. Detection of cytokine production by matured CBTC. Purified CB CD3⁺ T cells were matured with anti-CD3/-CD28 antibodies and rhIL-2. On day seven, cells were stimulated with PHA/PMA and incubated for 24h. Dead cells were excluded by gating on the FVS510 negative population and cytokine-producing cells were analysed by flow cytometry. The top panels represent the dot plots for IL-9, IL-21 cytokines, bottom panels represents the IFN-γ producing CBTC.

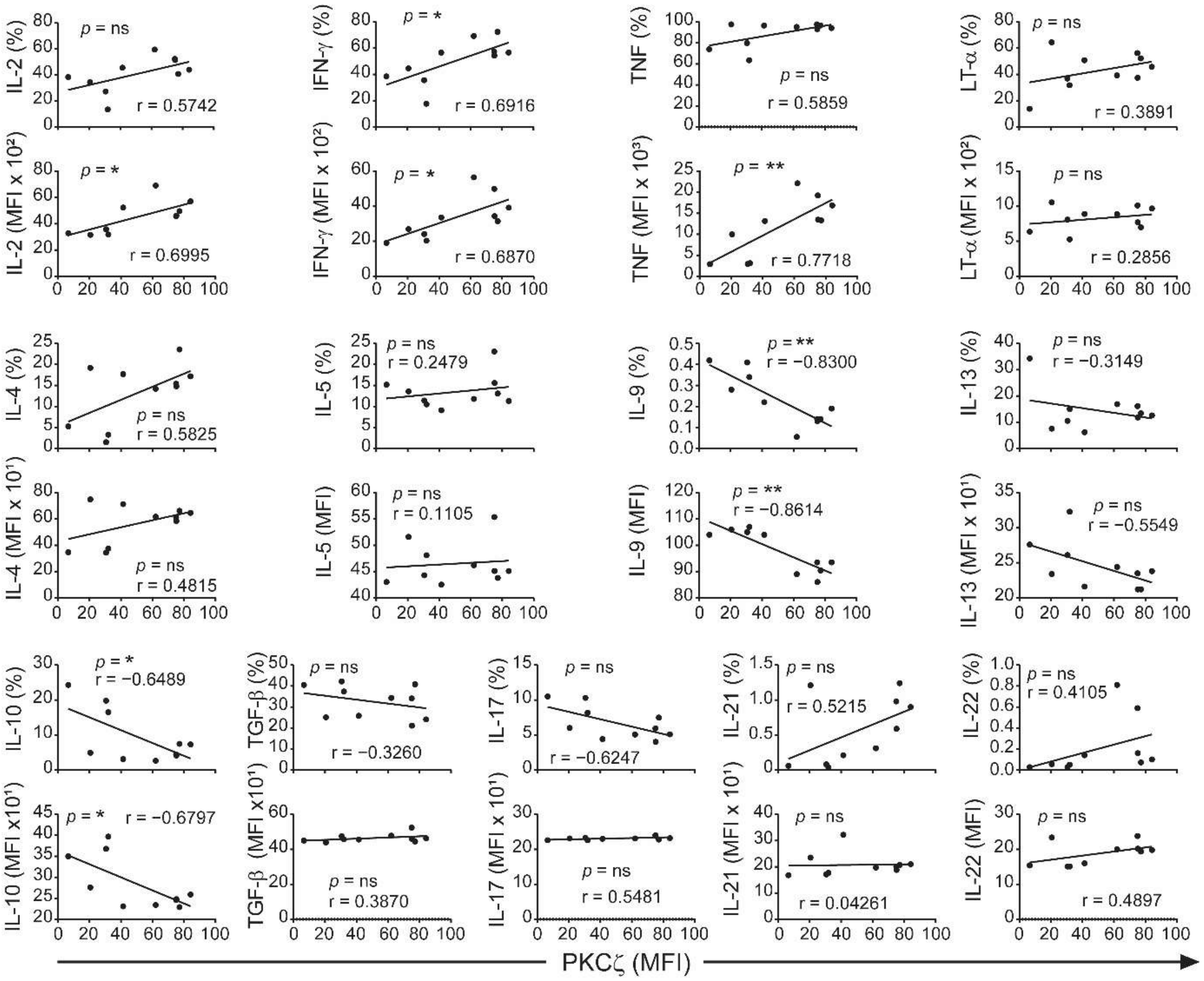

Figure S5. Correlation analyses of levels of PKCζ in CB $CD4^+$ T cells with cytokines produced by the matured T cells. Samples n=10. * $p < 0.05$, ** $p < 0.01$. ns: not significant. Correlations were performed using the two-tailed Pearson correlation.

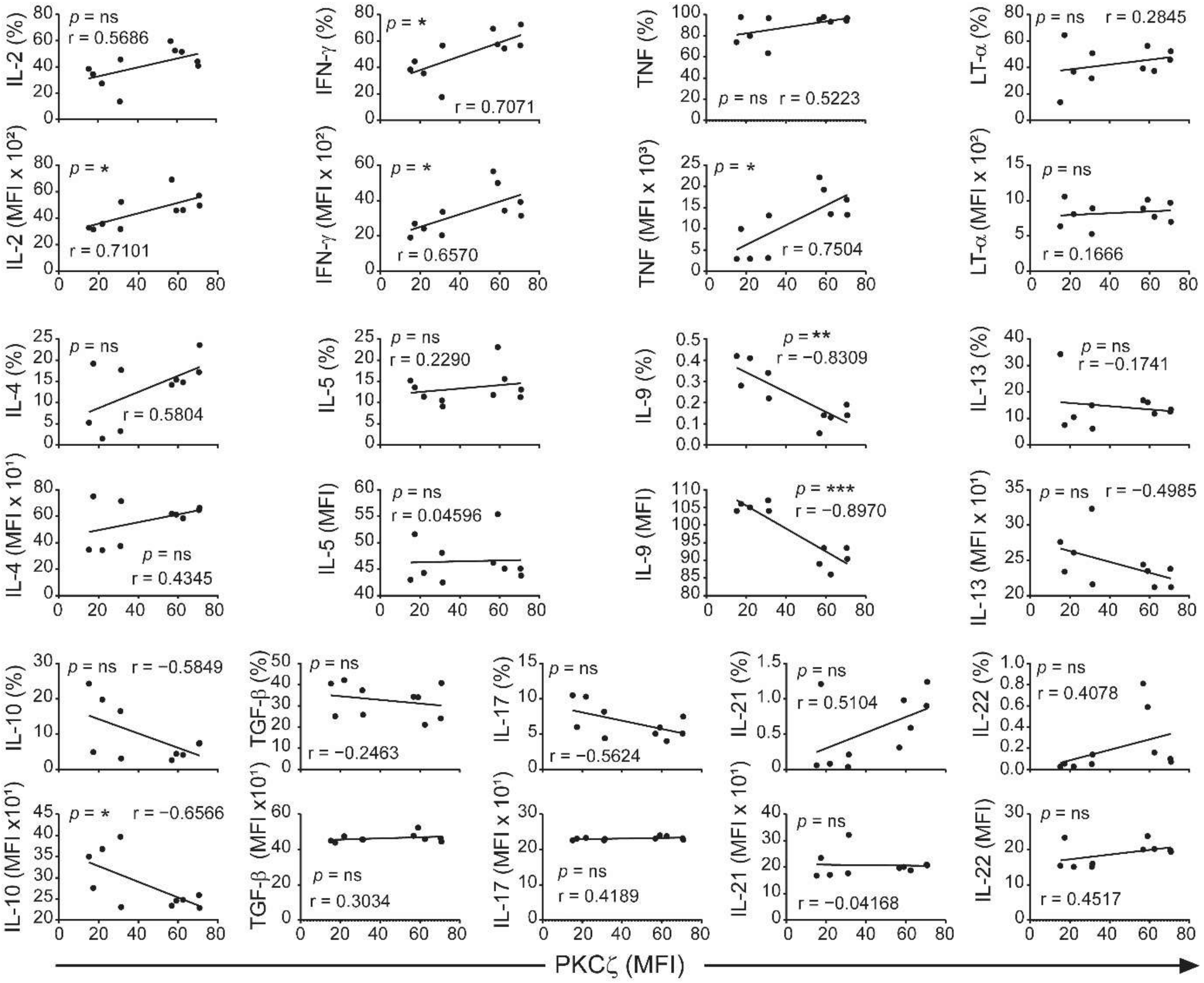

Figure S6. Correlation analyses of levels of PKCζ in CB CD8⁺ T cells with cytokines produced by the matured T cells. Samples n=10. * *p* < 005, ** *p* < 0.01. ns: not significant. Correlations were performed using the two-tailed Pearson correlation.
